# Cooperative Clinical Trial of Photodynamic Therapy for Early Gastric Cancer With Photofrin Injection^®^ and YAG-OPO Laser

**DOI:** 10.1155/DTE.4.165

**Published:** 1998

**Authors:** Seishiro Mimura, Hiroyuki Narahara, Toshio Hirashima, Hisayuki Fukutomi, Akira Nakahara, Hiromasa Kashimura, Hirofumi Matsui, Hiroshi Tanimura, Yugo Nagai, Shigeru Suzuki, Yoko Murata, Kazunari Yoshida, Kaichi Isono, Teruo Kozu, Hiroko Ide, Harubumi Kato

**Affiliations:** 1 Department of Gastroenterology Osaka Medical Center for Cancer and Cardiovascular Diseases 1-3-3 Nakamichi Higashinari-ku Osaka 537 Japan; 2 Department of Gastroenterology Chiba Nishi General Hospital Japan; 3 Department of lnternal Medicine University of Tsukuba Japan; 4 Second Department of Surgery Wakayama Medical School Japan; 5 Department of Gastrointestinal Endoscopy Tokyo Women's Medical College Japan; 6 Second Department of Surgery Chiba University Japan; 7 Department of Endoscopic Diagnostics and Therapeutics Chiba University Japan; 8 Department of Gastroenterology Tokyo Women's Medical College Japan; 9 Margins Department of Surgery Tokyo Medical College Japan

## Abstract

*Background and Objective*: Photodynamic therapy (PDT) treats malignant tumors using photosensitizers and light. We employed a new pulse laser as the excitation light source for PDT, i.e. an optical parametric oscillator (OPO) system pumped by a *Q*-switched Nd:YAG laser, because it provides extremely high peak power.

*Study Design/Materials and Methods*: The effects of PDT using the photosensitizer Photofrin^®^ and the new laser were evaluated in 12 patients with early gastric cancer.

*Results*: Complete responses (CR) were obtained in 75% of 12 assessable patients, CR was observed in all cases with mucosal carcinoma (response rate 100%).

Regarding toxicity, mild photosensitivity was seen in one case and it lasted several weeks. The other major side effect was decrease of total protein, which was observed in six patients (40%), lasting several months. There were no serious abnormalities in symptoms or laboratory tests.

*Conclusion*: We conclude that the YAG-OPO laser is suitable as an excitation light source for PDT.


*Diagnostic and Therapeutic Endoscopy*, Vol. 4, pp. 165–171
Reprints available directly from the publisher
Photocopying permitted by license only

© 1998 OPA (Overseas Publishers Association)
Amsterdam B.V. Published under license
under the Harwood Academic Publishers imprint,
part of The Gordon and Breach Publishing Group.
Printed in Singapore.

### Cooperative Clinical Trial of Photodynamic Therapy for Early Gastric Cancer with Photofrin Injection® and YAG-OPO Laser

SEISHIRO MIMURA[a,*], HIROYUKI NARAHARA[a], TOSHIO HIRASHIMA[b], HISAYUKI FUKUTOMI[c], AKIRA NAKAHARA[c], HIROMASA KASHIMURA[c], HIROFUMI MATSUI[c], HIROSHI TANIMURA[d], YUGO NAGAI[d], SHIGERU SUZUKI[e], YOKO MURATA[e], KAZUNARI YOSHIDA[e], KAICHI ISONO[f], TERUO KOZU[g], HIROKO IDE[h] and HARUBUMI KATO[i]

[a]*Department of Gastroenterology, Osaka Medical Center for Cancer and Cardiovascular Diseases, 1-3-3 Nakamichi, Higashinari-ku, Osaka 537, Japan;* [b]*Department of Gastroenterology, Chiba Nishi General Hospital;* [c]*Department of Internal Medicine, University of Tsukuba;* [d]*Second Department of Surgery, Wakayama Medical School;* [e]*Department of Gastrointestinal Endoscopy, Tokyo Women's Medical College;* [f]*Second Department of Surgery, Chiba University;* [g]*Department of Endoscopic Diagnostics and Therapeutics, Chiba University;* [h]*Department of Gastroenterology, Tokyo Women's Medical College;* [i]*Margins Department of Surgery, Tokyo Medical College*

*(Received 1 June 1997; In final form 29 September 1997)*

***Background and Objective:*** **Photodynamic therapy (PDT) treats malignant tumors using photosensitizers and light. We employed a new pulse laser as the excitation light source for PDT, i.e. an optical parametric oscillator (OPO) system pumped by a *Q*-switched Nd:YAG laser, because it provides extremely high peak power.**

***Study Design/Materials and Methods:*** **The effects of PDT using the photosensitizer Photofrin® and the new laser were evaluated in 12 patients with early gastric cancer.**

***Results:*** **Complete responses (CR) were obtained in 75% of 12 assessable patients, CR was observed in all cases with mucosal carcinoma (response rate 100%).**

**Regarding toxicity, mild photosensitivity was seen in one case and it lasted several weeks. The other major side effect was decrease of total protein, which was observed in six patients (40%), lasting several months. There were no serious abnormalities in symptoms or laboratory tests.**

***Conclusion:*** **We conclude that the YAG-OPO laser is suitable as an excitation light source for PDT.**

*Keywords:* Endoscopic treatment, YAG-OPO laser, Hematoporphyrin derivative (HpD), Photodynamic therapy (PDT), Early gastric cancer

* Corresponding author.

### INTRODUCTION

Photodynamic therapy (PDT) is a relatively recently developed endoscopic method for treating malignant tumors using a photosensitizer, initially hematoporphyrin derivative (HpD), and a laser as the excitation light source [1,2]. The principle of this method is to kill malignant cells by photo-chemical reaction rather than heat. Because HpD has a higher affinity for malignant tissue than normal tissue, after intravenous injection it is taken up and retained longer by malignant tissue [3]. Thus by using weak laser light to irradiate the tumor region, malignant tissue can be destroyed selectively [4]. Photofrin Injection® (PHE) which became commercially available in May 1995 in Japan, is freeze-dried Photofrin II manufactured by American Cyanamid Co., New York, and is imported by Lederle (Japan) Co., Ltd.

Recently several new kinds of photosensitizers such as benzoporphyrin derivative (BPD), meta-tetrahydroxyphenyl chlorin (mTHPC), mono-L-aspartyl chlorin (NPe6) and tin ethyl etiopurpurin (ZnET2) have attracted attention because they are all superior to PHE in purity, have higher affinity for malignant tumors and show strong absorption at longer wavelengths. Clinical studies on these new drugs have been performed in several countries.

Laser equipment such as the argon dye laser, nitrogen dye laser, gold vapor laser, copper vapor dye laser and excimer dye laser have also been developed, and have been used clinically in Japan. Among these the excimer dye laser (EDL, model PDT EDL-1, Hamamatsu Photonics, Hamamatsu, Japan) was authorized as a light source for PDT by the Ministry of Welfare of Japan in 1994 [5]. The indications of PDT employing PHE and the EDL are limited to early stage lung cancer, early stage esophageal cancer, early stage gastric cancer and early stage cancer and dysplasia of the uterine cervix. In the EDL an ultraviolet laser beam of 308 nm wavelength, which is produced by the XeCl excimer laser, pumps the dye laser with Rhodamine 640, which then emits a secondary laser beam of 630 nm wavelength when the concentration of the dye is adjusted to 0.4 mM. The EDL has the following characteristics: wavelength, 630 nm; pulse energy, 4 mJ; peak power, 400 kW; pulse width, 10 ns; frequency of repetition: 20, 30 or 40 Hz [6]. It was demonstrated in animal tumors [7], in clinical studies on gastric cancer [8], and also theoretically [9] that a pulse laser with an extremely high peak power is superior to a continuous wave laser in terms of the depth of photodynamic action. However, the structure of the EDL is complex and physicians feel it is difficult to use, because it needs exchange of helium gas in the excimer laser and the dye solution in the dye laser. Moreover, it is clear that tunability to longer wavelengths for new photosensitizers will be required in the near future. We therefore carried out a clinical study using a new laser as a light source for PDT.

### MATERIALS AND METHODS

### Eligibility Criteria

Eligibility criteria included (1) biopsy-proven early gastric cancer that was evaluated as mucosal or submucosal invasion, (2) either less than 3 cm in diameter or 7 $cm^2$ tumor area, (3) entire lesion visible endoscopically, (4) no prior therapy for the targeted tumor lesion, however residual lesions after endoscopic treatments other than PDT were eligible, (5) informed consent of either patients or their relatives before starting therapy. There was no restriction on the age of patients.

### Photosensitizer

PHE, which is purified freeze-dried hematoporphyrin derivative for intravenous use, contains 75 mg/vial porfimer sodium. The active ingredient, porfimer sodium, consists of a mixture of porphyrin oligomers of up to eight porphyrin units. The main ingredient is dihematoporphyrin ether/ester.

### Laser Equipment

The laser equipment employed in this trial was an optical parametric oscillator system pumped by a $Q$-switched Nd:YAG laser (YAG-OPO laser, model iLS-TL-50A, Ishikawajima-Harima Heavy Industries Co., Ltd. (IHI) Tokyo, Japan). This laser system consists of a $Q$-switched Nd:YAG laser that emits a pulse laser beam coupled to an optical parametric oscillator system. The 1064 nm wavelength laser beam of the $Q$-switched Nd:YAG laser is pumped by a flash lamp and is converted into a third harmonic generation (THG) of 355 nm wavelength through two kinds of nonlinear crystals. The THG pumps an OPO composed of one set of oscillators and one crystal. Consequently, two kinds of laser beams are generated, one is the signal, and the other is the idler. The wavelengths of the two laser beams can be changed by tuning the angle of incidence into the OPO. One of these two beams is used for photoradiation in PDT. Major specifications of this system are (1) laser wavelength: 620–670 nm, tuned to 630 nm in this trial; (2) pulse energy: maximum 6 mJ, energy used was 5 mJ through 400 µm core diameter quartz fiber; (3) peak power: between 700 kW and 1 MW; (4) pulse width: 5–8 ns; and (5) pulse frequency: 25 or 50 Hz, 50 Hz was used in this trial [10].

### PDT Procedure

Patients were intravenously given 2.0 mg/kg of PHE after 75 mg/vial of PHE was dissolved in 30 ml of 5% glucose and photoradiation was carried out 48–53 hours later. The entire lesion plus about a 5 mm wide margin was irradiated with the YAG-OPO laser beam transmitted endoscopically. When the distance between the tumor surface and the fiber tip was 3.1 cm and the diversion angle of the laser beam was 20.5°, the irradiation area was approximately 1 $cm^2$. The irradiation was delivered at a total energy intensity of more than 60 $J/cm^2$. For wider lesions, the irradiation field was first set on a part of the lesion, and after delivering the scheduled dose of light there, the field of irradiation was shifted to the remaining part, in order to irradiate the entire region as uniformly as possible. Two hundred forty seconds of irradiation were required to obtain 60 J with 5 mJ of pulse energy, and 50 Hz of pulse frequency. Assuming that the irradiation was uniform, the total energy intensity ($J/cm^2$) was calculated by the following formula:

$$\begin{aligned}\text{Total energy intensity } (J/cm^2) &= \text{pulse energy } (J/\text{pulse}) \\ &\quad \times \text{pulse frequency (pulse/s)} \\ &\quad \times \text{time(s)/total irradiated area } (cm^2)\end{aligned}$$

After PDT, the patients were given $H_2$-receptor antagonist as prophylactic treatment of the ulcer that would develop. For safety, patients were exposed as little as possible to direct sunlight for at least 4 weeks after PDT, and were recommended to wear a wide-brimmed hat, sunglasses, sunscreen lotion, long-sleeved shirts, and gloves.

### Evaluation of Response

The response to PDT was evaluated by endoscopy and biopsy 1, 2, and 4 weeks, and 2, 3, and 6 months, and 1 year after PDT, and every 6 months thereafter. Tumor response to treatment was evaluated as complete response (CR): no evidence of tumor either histologically or endoscopically for at least 4 weeks, partial response (PR): more than 50% reduction in size of tumor for at least 4 weeks, and no change (NC): no change in size of tumor. Drugs other than PHE that would affect tumor response or adverse effects such as anticancer drugs were prohibited during PDT except for drugs for the treatment of adverse effects.

### RESULTS

### Patient Characteristics

Fifteen patients were entered from June 1995 through December 1996. Of these 15 patients, 12 were evaluable for response. One ineligible patient had a lesion of more than 7 $cm^2$. One patient dropped out, because the lesion was judged to be an advanced gastric cancer by the study committee. One nonevaluable patient was treated by microwave coagulation one week after PDT.

The characteristics of the 12 eligible patients are summarized in Table I. The median age was 68.5 years old, and the median performance status (ECOG) was 0 (Karnofsky performance status 100%). Five patients' lesions were located in the upper third, six in the middle third, and one in the lower third of the stomach. Nine patients had well-differentiated tubular adenocarcinoma, one moderately differentiated tubular adenocarcinoma, one poorly differentiated adenocarcinoma and one signet-ring cell carcinoma. Median tumor area was 3.8 $cm^2$. Four had mucosal carcinomas and eight submucosal carcinomas. Three had superficial elevated type, two superficial depressed type without ulceration, and seven superficial depressed type with ulceration of early gastric cancer. Eleven patients were ineligible for laparotomy, because of the liver, lung, cardiovascular or kidney function in 10 patients and old age in one patient. One patient preferred PDT, because a small cancer nest was left behind after an endoscopic mucosal resection at another institute, and the depth of invasion had been proved to be mucosal carcinoma in the resected specimen.

TABLE I Characteristics for eligible cases

| | |
|---|---|
| Sex | |
| Male | 10 |
| Female | 2 |
| Age (years) | |
| 50–59 | 1 |
| 60–69 | 7 |
| 70–79 | 3 |
| 80–89 | 1 |
| Performance status (EOCG) | |
| 0 | 9 |
| 1 | 2 |
| 2 | 1 |
| 3 | 0 |
| 4 | 0 |
| Tumor location | |
| Upper third | 5 |
| Middle third | 6 |
| Lower third | 1 |
| Histology | |
| Wel. diff. tubular adenocarcinoma* | 9 |
| Mod. diff. tubular adenocarcinoma† | 1 |
| Poorly diff. adenocarcinoma‡ | 1 |
| Signet-ring cell carcinoma | 1 |
| Tumor area ($cm^2$) | |
| –1.0 | 0 |
| 1.1–2.0 | 1 |
| 2.1–4.0 | 6 |
| 4.1–7.0 | 5 |
| Depth of invasion | |
| Mucosal | 4 |
| Submucosal | 8 |
| Gross type | |
| Superficial elevated | 3 |
| Superficial depressed Ul(–)¶ | 2 |
| Superficial depressed Ul(+)§ | 7 |

* Well-differentiated tubular adenocarcinoma
† Moderately differentiated tubular adenocarcinoma
‡ Poorly differentiated adenocarcinoma
¶ Without ulceration
§ With ulceration

CR was obtained in 9 of 12 patients (75%), 7 by the initial PDT, while two required a second PDT to obtain CR. Three PR cases had characteristics such as submucosal invasion, tumor area more than 2.1 $cm^2$ in two cases, and tumor area more than 4.1 $cm^2$ in one case. The 9 CR patients had no relapse between 4 and 20 months, with a median of 9 months. There were no particular patient characteristics which seemed to influence the CR rate, but patients with mucosal carcinoma had a 100% CR rates as shown in Table II.

### Toxicity

Toxicity was evaluated in all 15 patients who entered the study. The results are summarized in Table III. The main abnormality on laboratory tests was decrease of total protein observed in six patients (40%), who all recovered within several months with normal diet, except one patient with liver cirrhosis who required intravenous injection at 150 ml of 25% human albumin. Photosensitivity, observed in one patient, lasted several weeks. Decrease of erythrocyte and hemoglobin level lasted several months in two patients. All these toxicities were grade 1 or slight. No serious adverse reactions were seen.

TABLE II Patient characteristics and response

| Characteristics | CR | PR | Relapse in CR |
|---|---|---|---|
| Sex | | | |
| Male | 7 | 3 | 0 |
| Female | 2 | 0 | 0 |
| Age (years) | | | |
| 50–59 | 1 | 0 | 0 |
| 60–69 | 4 | 3 | 0 |
| 70–79 | 3 | 0 | 0 |
| 80–89 | 1 | 0 | 0 |
| Performance status (EOCG) | | | |
| 0 | 7 | 2 | 0 |
| 1 | 1 | 1 | 0 |
| 2 | 1 | 0 | 0 |
| 3 | 0 | 0 | 0 |
| 4 | 0 | 0 | 0 |
| Tumor location | | | |
| Upper third | 4 | 1 | 0 |
| Middle third | 4 | 2 | 0 |
| Lower third | 1 | 0 | 0 |
| Histology | | | |
| Wel. diff. adenocarcinoma* | 8 | 1 | 0 |
| Mod. diff. adenocarcinoma† | 0 | 1 | 0 |
| Poorly diff. adenocarcinoma‡ | 1 | 0 | 0 |
| Signet-ring cell carcinoma | 0 | 1 | 0 |
| Tumor area ($cm^2$) | | | |
| −1.0 | 0 | 0 | 0 |
| 1.1–2.0 | 1 | 0 | 0 |
| 2.1–4.0 | 4 | 2 | 0 |
| 4.1–7.0 | 4 | 1 | 0 |
| Depth of invasion | | | |
| Mucosal | 4 | 0 | 0 |
| Submucosal | 5 | 3 | 0 |
| Gross type | | | |
| Superficial elevated | 2 | 1 | 0 |
| Superficial depressed Ul(−)¶ | 1 | 1 | 0 |
| Superficial depressed Ul(+)§ | 6 | 1 | 0 |

*Well-differentiated tubular adenocarcinoma
†Moderately differentiated tubular adenocarcinoma
‡Poorly differentiated adenocarcinoma
¶Without ulceration
§With ulceration

TABLE III Side effects

| | Incidence | Grade | | | |
|---|---|---|---|---|---|
| | (%) | 1 | 2 | 3 | 4 |
| Photosensitivity* | 1 (6.7) | 1 | | | |
| Erythrocytopenia† | 2 (13.3) | | | | |
| Low hemoglobinemia* | 2 (13.3) | 2 | | | |
| Hypoproteinemia† | 6 (40) | | | | |

*WHO grade
†Not graded

### DISCUSSION

### Efficacy of PDT Using PHE and YAG-OPO Laser

In 1996, one of the authors (S. M.) reported the results of a cooperative clinical trial of PDT for early gastric cancer in 27 patients using PHE and an EDL [11]. A CR rate of 88% was obtained, that is 21 out of 24 patients evaluable for response, but among the 21 CR, three cases relapsed. The rate of CR without relapse therefore was 75%, i.e. 18 out of 24, while that of this present study was also 75%, i.e. 9 out of 12 patients evaluable for response. However, according to the tumor size, the former study included 10 patients with lesions smaller than 1 $cm^2$, all of whom CR was obtained, while this study had no cases with such small lesions. Comparing results for lesions larger than 2.1 $cm^2$, the rate of CR without relapse was 58%, i.e. 7 out of 12 in the former study with the EDL, while it was 73%, i.e. 8 out of 11 in the present study using the YAG-OPO laser. Briefly, the CR rates without relapse in both studies were similar, but in lesions larger than 2.1 $cm^2$, there was a tendency for the YAG-OPO laser to be slightly superior to the EDL as a light source in PDT.

### Side Effects

Although the main symptoms reported in the literature were skin reactions such as photosensitivity, edema and pigmentation, in the present trial the only case of facial edema had basked in direct sunlight 3 weeks after administration of PHE. Observance of our instructions, which recommend avoidance of direct sunlight for 4 weeks should prevent such side effects. Another major side effect was decrease of total protein, which was first reported in 1985 [12], and was thought to be caused by protein loss from the base of the ulcer that developed after PDT and acute gastritis surrounding the ulcer. The wider the lesion treated PDT, the greater the likelihood of a problem, especially in the cases with a tendency toward hypoalbuminemia before treatment.

### Clinical Use of PDT

Surgery is the first treatment of choice for early gastric cancer. However, although more early stage cases are now being detected as a result of improvements in survey and diagnostic techniques, many patients are at high surgical risk due to coexisting hepatic, cardiovascular and/or pulmonary diseases. For these patients, endoscopic treatment is desirable. Several kinds of methods have been developed for the endoscopic treatment of early gastric cancer, such as endoscopic mucosectomy, but none of them is effective for treatment of the depressed type with ulceration and lesions with submucosal invasion. In the present trial using PHE and a YAG-OPO laser, the rate of CR without relapse was 86% (6/7) in patients with superficial depressed type with ulceration in which other endoscopic treatments were not indicated. Furthermore, in patients with carcinoma invading the submucosal layer, the rate of CR without relapse was 63% (5/8). These results are promising for the treatment of superficial depressed type lesions with ulceration, and carcinoma invading the submucosal layer.

Concerning lesion size, eligibility criteria in the present study required either less than 3 cm in diameter or 7 $cm^2$ of tumor area, based on a former study using the EDL. However, in one case of a 79-year-old man, who was ineligible because of tumor size, CR was achieved with no relapse. The lesion extended over 19 $cm^2$ in the mucosal layer, located in the middle third of the stomach on the lesser curvature, the gross type was superficial elevated type, and the histologic diagnosis was well-differentiated tubular adenocarcinoma. For this lesion, photoradiation with a YAG-OPO laser was performed at 53 h after administration of PHE, giving 1080 J for 27 $cm^2$, which was calculated as 40 $J/cm^2$ of total energy intensity. This result suggests two points, one is that PDT can cure an extensive lesion spreading over 19 $cm^2$, and the other is that an energy intensity of only 40 $J/cm^2$ can obtain CR in mucosal cancer. The former will increase the indications of PDT for more extensive lesions, and the latter will also expand it, because the lower the energy intensity required, the greater the area that can be treated within a certain period of time.

The above results suggest that PDT is indicated in cases of superficial depressed carcinoma including lesions with ulceration. However, the solitary elevated type can be easily treated by other endoscopic methods. In addition, PDT is indicated in cases of carcinoma with submucosal invasion and it is indicated for patients who are at poor risk for surgery. Finally, PDT is also indicated in cases with relatively large superficial lesions.

### *References*

[1] Dougherty, T.J., Lawrence, G., Kaufman, J.H., Boyle, D., Weishaupt, K.R. and Goldfarb, A.: Photoradiation in the treatment of recurrent breast carcinoma. *J. Natl. Cancer Inst.* 1979; **62**: 231–237.

[2] Hayata, Y., Kato, H., Konaka, C., Ono, J. and Takizawa, N.: Hematoporphyrin derivative and laser photoradiation in the treatment of lung cancer. *Chest* 1982; **81**: 269–277.

[3] Lipson, R.L. and Baldes, E.J.: The photodynamic properties of a particular hematoporphyrin derivative. *Arch. Dermatol.* 1960; **82**: 508–516.

[4] Mimura, S., Ichii, M., Imanishi, K., Tatsuta, M., Otani, T. and Okuda, S.: Evaluation of photodynamic therapy. *Dig. Endosc.* 1990; **2**: 265–274.

[5] Hayata, Y., Kato, H., Huruse, K., Kusunoki, Y., Suzuki, S. and Mimura, S.: Photodynamic therapy of 168 early stage cancers of the lung and oesophagus: a Japanese multi-centre study. *Lasers Med. Sci.* 1996; **11**: 255–259.

[6] Hirano, T., Ishizuka, M., Suzuki, K. *et al*: Photodynamic cancer diagnosis and treatment system consisting of pulsed lasers and an endoscopic spectro-image analyzer. *Lasers Life Sci.* 1989; **3**: 99–116.

[7] Okunaka, T., Kato, H., Konaka, C., Sakai, H., Kawabe, H. and Aizawa, K.: A comparison between argon-dye and excimer-dye laser for photodynamic effect in transplanted mouse tumor. *Jpn. J. Cancer Res.* 1992; **83**: 226–231.

[8] Mimura, S., Ichii, M. and Okuda, S.: Photodynamic therapy for early gastric cancer using excimer dye laser. *Photodynamic Therapy and Biomedical Lasers*, Spinelli, P., Dal Fante, M. and Marchesini, R., Editors, pp. 272–276, Elsevier Science Publishers B. V., Amsterdam, 1992.

[9] Takemura, T., Umeuchi, S., Nakajima, S. and Sakata, I.: "Mechanism of photodynamic therapy (PDT): investigation of sensitizer dose and light dose-rate effects," in *5th International Photodynamic Association Biennial Meeting*, Denis A. Cortese, Editor, *Proc. SPIE* **2371**, 351–354, 1995.

[10] Udagawa, T., Inoue, K., Fukutomi, S. and Takaoka, K.: YAG-OPO laser for PDT application. *J. Jpn. Soc. Laser Med.* 1995; **16**(3): 25–30.

[11] Mimura, S., Ito, Y., Nagayo, T. *et al.*: Cooperative clinical trial of photodynamic therapy with Photofrin II and excimer dye laser for early gastric cancer. *Lasers Surg. Med.* 1996; **19**: 168–172.

[12] Mimura, S., Ichii, M., Sato, M. and Okuda, S.: Photodynamic therapy for early gastric cancer, the method of irradiation and medication for patients. *Med. Consult. New Remed.* 1985; **22**: 1351–1363.

